# Identification of Circular RNAs Circ_0005008 and Circ_0005198 in Plasma as Novel Biomarkers for New-Onset Rheumatoid Arthritis

**DOI:** 10.3389/fphar.2021.722017

**Published:** 2021-09-01

**Authors:** Qingqing Ouyang, Chixiang Liu, Xiaoxi Lu, Renge Liang, Jinjun Zhao, Min Yang

**Affiliations:** ^1^ Department of Rheumatology and Immunology, Nanfang Hospital, Southern Medical University, Guangzhou, China; ^2^ Transfusion Department, Nanfang Hospital, Southern Medical University, Guangzhou, China

**Keywords:** rheumatoid arthritis, circular RNAs, microarray assay, fibroblast-like synoviocytes, biomarker

## Abstract

The progression of autoimmune diseases is affected by the differential expression of circular RNAs (circRNAs). However, in the plasma from rheumatoid arthritis (RA), circRNAs have an uncertain role. Herein, microarray analysis was used to determine the plasma expression profile of circRNAs from new-onset patients with RA and healthy controls (HCs). CircRNA expression was verified using quantitative real-time reverse transcription PCR. The correlation between clinical variables and circRNA expression was assessed using Spearman’s correlation test. The diagnostic value of plasma circRNAs was evaluated using receiver operating characteristic (ROC) curves. Circ_0005008 and circ_0005198 were confirmed to be elevated significantly in plasma samples from new-onset patients with RA compared with those from HCs and from patients with systemic lupus erythematosus. Among these new-onset patients with RA, we found that the levels of circ_0005008 and circ_0005198 correlated positively with the severity of disease, including the rheumatoid factor, C-reactive protein, the erythrocyte sedimentation rate, and the disease activity score in 28 joints (DAS28). However, their expression levels did not correlate with anti-cyclic citrullinated peptide antibodies. Analysis using ROC curves implied that circ_0005008 and circ_0005198 have significant value in the diagnosis of RA. In addition, we found that compared with that in osteoarthritis fibroblast-like synoviocytes (OA-FLSs), circ_0005198 expression was enhanced in RA-FLSs and correlated positively with DAS28. The level of the miRNA target of circ_0005198, miR-4778-3p, was identified as significantly decreased in RA-FLSs, and the expression levels of circ_0005198 and miR-4778-3p correlated significantly and negatively. The results suggested that in new-onset patients with RA, plasma circ_0005008 and circ_0005198 levels are associated with disease activity and represent possible RA biomarkers.

## Introduction

The hallmarks of the chronic systemic autoimmune disease, rheumatoid arthritis (RA), are joint destruction, synovitis, and the manifestation of systemic immunity and inflammation. Although patients with RA can experience improved treatment and survival, the majority continue to suffer from long-term joint damage, severe illness, and lifelong disability (Boutet et al., 2021). Early diagnosis and appropriate treatment are considered to contribute to the prevention of serious disease symptoms in patients with RA. However, the specific mechanisms causing RA development and progression are not clear (Chen et al., 2016).

The recently discovered noncoding RNAs, circular RNAs (circRNAs), mainly comprise exon-derived transcripts that are produced via non-colinear reverse splicing. CircRNAs are expressed widely in various human cells and have crucial functions in post-transcriptional gene expression regulation (Capel et al., 1993; Memczak et al., 2013). Accumulating evidence indicates that in various diseases, circRNAs could be used as biomarkers for early diagnosis and prognosis prediction (Wen et al., 2020; Chen et al., 2021). Hsa_circ_0044235 levels in peripheral blood could represent a biomarker for patients with RA (Luo et al., 2018). Previously, we showed that RA might be diagnosed using the circRNA levels in peripheral blood mononuclear cells from patients (Ouyang et al., 2017). In addition, in patients with lupus nephritis, diagnosis might be achieved using plasma circRNA_002453 as a biomarker (Ouyang et al., 2018). However, we lack knowledge of levels of these circRNAs in plasma from new-onset patients with RA.

Therefore, we aimed to ascertain if plasma circRNAs have value as biomarkers in new-onset patients with RA. Investigation of the dysregulated expression levels of circRNAs and their mechanisms in RA might lead to a deeper understanding of the development of RA and the identification of new therapeutic strategies.

## Materials and Methods

### Characteristics of the Patients

In this study, we recruited 121 participants, comprising 49 patients with RA, 25 patients with systemic lupus erythematosus (SLE), 40 healthy controls (HCs), and seven patients with osteoarthritis (OA). Diagnosis of OA, SLE, and RA were carried out at the Department of Rheumatology and Immunology at Nanfang Hospital of the Southern Medical University from 2015 to 2017. The recruited HCs comprised sex and aged-matched healthy people who received regular physical examinations at the Department of Health at the above hospital. All participants satisfied the criteria from the American College of Rheumatology for the classification of RA (Aletaha et al., 2010), OA (Altman et al., 1986), or SLE (Aringer et al., 2019). During total hip replacement surgery (carried out at the Department of Orthopedic Surgery, Nanfang Hospital, Southern Medical University, Guangzhou, China), nine RA synovial tissues and seven OA synovial tissues were sampled from patients with end-stage symptomatic hip RA or OA. New-onset patients with RA or SLE who provided the plasma sample suffering from diabetes mellitus or malignancies, or diagnosed to have overlapping syndromes (i.e., coexisting connective tissue diseases, including scleroderma or Sjogren’s syndrome), or with serious liver or kidney diseases, or receiving any type of nonsteroidal anti-inflammatory drug, DMARD, glucocorticoid, immunesuppressor, or biological agent were not included in this study. The DAS28 scores were defined as follows: remission (score <2.6), low/moderate disease activity (score 2.6 to ≤5.1) and very active disease (score >5.1) (Singh et al., 2016). The ethics committee of Nanfang Hospital of Southern Medical University approved the study protocols (NO. NFEC-2015-102 and NFEC-20120201). All participants provided written informed consent.

### Total RNA Extraction From Prepared Plasma

Blood samples (approximately 8 ml) were collected into tubes treated with EDTA. To collect the plasma, the blood samples were centrifuged immediately at 3,000 × g at 4°C for 15 min. The plasma was placed at −80°C until further use. Plasma (200 μl) was subjected to total RNA isolation using an miRNeasy Serum/Plasma Kit (Qiagen, Hilden, Germany) following the manufacturer’s protocol. The TRIzol reagent (Takara, Shiga, Japan) was used to acquire total RNA from cultured cells following the manufacturer’s guidelines.

### Microarray Hybridization

Following the manufacturer’s protocols (Arraystar Inc., Rockville, MD, United States), the samples were labeled and hybridized to the microarray. In brief, RNase R (Epicentre Inc., Madison, WI, United States) digestion of the total RNA was used to enrich circRNAs by removing linear RNAs. In the Arraystar Super RNA Labeling Kit, a random priming method was used to amplify and transcribe the enriched circRNAs into fluorescently-labeled circRNAs. Then, the labeled circRNAs were hybridized to the Arraystar Human circRNA Microarray version 2.0 (8 × 15 K). The slides were washed, and then an Agilent G2505C Scanner (Agilent, Santa Clara, CA, United States) was used to scan the arrays. The acquired array images were analyzed using Agilent Feature Extraction software (version 11.0.1.1). The R software package (R version 3.1.2) was then used for quantile normalization and subsequent data processing. KangChen Bio-tech (Shanghai, People’s Republic of China) carried out the microarray experiment.

### Quantitative Real-Time Reverse Transcription PCR Analysis

The prepared total RNA was subjected to reverse transcription to produce cDNA. Subsequently, the cDNA was used as the template in the qPCR step, which used SYBR Premix DimerEraser (Takara) in an ABI7500 system (Applied Biosystems, Foster City, CA, United States). For the circRNAs, the internal control was ACTB (encoding β-actin) and for the miRNAs, the internal control was U6. The primers used for qRT-PCR are shown in Supplementary Table S1. The 2-ΔΔCt method (Livak and Schmittgen, 2001) was used to analyze the data, which are reported as relative expression levels from three independent experiments.

### Culture of Fibroblast-Like Synoviocytes

Human synovial tissue specimens were used to isolate FLSs, which were cultured using Dulbecco’s modified Eagle’s medium (DMEM) (Thermo Fisher Scientific, Inc., Waltham, MA, United States) containing 10% fetal bovine serum (FBS) (Gibco BRL, Grand Island, NY, United States). Three to five passages of FLSs were performed before they were used. Vimentin immunofluorescence staining was used to confirm that the RA-FLSs were a homogenous population with a purity >98% (Supplementary Figure S1).

### Immunofluorescent Staining

4% paraformaldehyde was used to fix the RA-FLSs for 20 min, and then 0.2% Triton X-100 was used to permeabilize them at room temperature for 5 min. After washing, 10% normal goat serum (BioSS, Beijing, China) was added to block the cells at room temperature for 1 h. The cells were then incubated overnight at 4°C with antibodies against vimentin (1:100). After three washes with phosphate buffered saline, the cells were incubated at room temperature for 1 h with goat anti-rabbit IgG/Alexa Fluor 647 antibodies (1:200). 4′,6-diamidino-2-phenylindole (DAPI) was used to counterstain the nuclei. A Leica TCS SP2 AOBS confocal microscope (Leica Microsystems, Wetzlar, Germany) was then used to acquire images.

### Statistical Analysis

Student’s *t*-test or the Mann-Whitney test, as appropriate, were used to evaluate the statistical significance among the groups. Spearman’s rank correlation was used to analyze the associations between parameters. To assess the diagnostic utility of dysregulated plasma circRNAs from patients with RA compared with those in the controls (HCs and patients with SLE), we created receiver operating characteristic (ROC) curves. *p* < 0.05 was accepted to indicate statistical significance. SPSS version 16.0 (IBM Corp. Armonk, NY, United States) was used to carry out all the statistical analyses.

## Results

### Screening of the Abnormal Expression CircRNAs in Plasma From New-Onset Patients With RA and HCs

**FIGURE 1:**
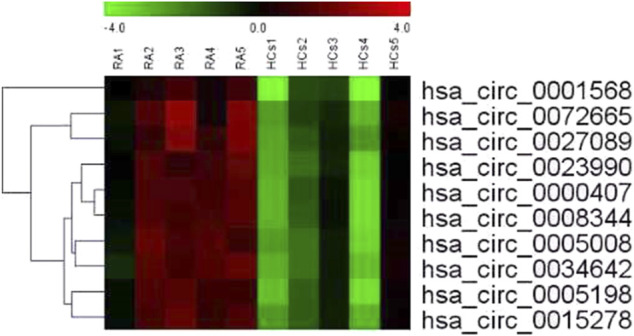
Cluster analysis of the comparison of 10 circRNAs that showed differential expression in 5 new-onset patients with RA compared with their expression levels in 5 HCs. Each row represents an different gene; each column represents an individual sample. “Red” marks high relative expression and “green” marks low relative expression (readers are referred to the web version of this article to see the interpretation of the colors in this figure).

Table 1 shows the characteristics of the study participants at baseline. Plasma circRNAs from five new-onset patients with RA and five age- and sex-matched HCs were subjected to microarray analysis using the Arraystar Human circRNA Microarray version 2.0 to identify those circRNAs that are differentially expressed in RA. The raw data were normalized, followed by screening using the criteria of log2 fold-changes greater than 5 and *p* < 0.05, which identified 10 circRNAs that were significantly differentially expressed (see Figure 1 for the cluster analysis results). Compared with their expression levels in HCs, 10 circRNAs were all upregulated in new-onset patients with RA (Table 2).

**TABLE 1:** Clinical features and laboratory information of the participants.

	Microarray analysis		Validation			
	RA	Control	RA	SLE	Control	OA
	(*n* = 5)	(*n* = 5)	(*n* = 49)	(*n* = 25)	(*n* = 40)	(*n* = 7)
Age (years)	45 (35–65)	48 (32–68)	51 (17–71)	28 (16–40)	50 (30–66)	50 (49–54)
Sex (M/F) (*n*)	2/3	2/3	18/31	7/18	14/26	2/5
CRP (mg/L)	65.2 (25.3–91.1)	NA	23.8 (0.34–107.3)	25.2 (0.1–113.5)	NA	5 (0.5–10)
ESR (mm/1 h)	59 (41–95)	NA	38 (2.2–112)	40 (9–102)	NA	15 (7–23)
IgM RF(IU/mL)	98.8 (60–411)	NA	154.5 (9.19–4,700)	NA	NA	NA
Anti-CCP (U/mL)	83.4 (30.1–450)	NA	133.7 (7.8–1,200)	NA	NA	NA
DAS28 (scores)	5.74 (5.08–7.25)	NA	5.82 (3.08–8.16)	NA	NA	NA

**Abbreviations:** RA: rheumatoid arthritis; SLE: systemic Lupus Erythematosus; OA: osteoarthritis; M/F: male/female; ESR: erythrocyte sedimentation rate; CRP: C-reactive protein; DAS28: disease activity score in 28 joints; RF: rheumatoid factor; anti-CCP: anti-cyclic citrullinated peptide antibodies; NA: not available. Data are expressed as the median (range).

**TABLE 2:** Differentially expressed circRNAs in plasma from new-onset patients with RA and healthy controls.

CircRNA	*p*-value	Fold change	Mode of regulation	Gene symbol
hsa_circ_0001568	0.019669755	6.8932736	up	DUSP22
hsa_circ_0023990	0.004710925	6.2519308	up	NOX4
hsa_circ_0005008	0.008125146	6.0257775	up	HNRNPA1P48
hsa_circ_0000407	0.008819279	6.0222696	up	SMARCC2
hsa_circ_0008344	0.010327312	6.0167564	up	UBAP2
hsa_circ_0005198	0.010271275	5.9127625	up	PARP4
hsa_circ_0034642	0.010766813	5.8900901	up	VPS18
hsa_circ_0072665	0.013482826	5.8529225	up	ADAMTS6
hsa_circ_0027089	0.00682063	5.6921968	up	PTGES3
hsa_circ_0015278	0.006022201	5.1897478	up	KLHL20

### CircRNAs Expression Validation Using qRT-PCR

**FIGURE 2:**
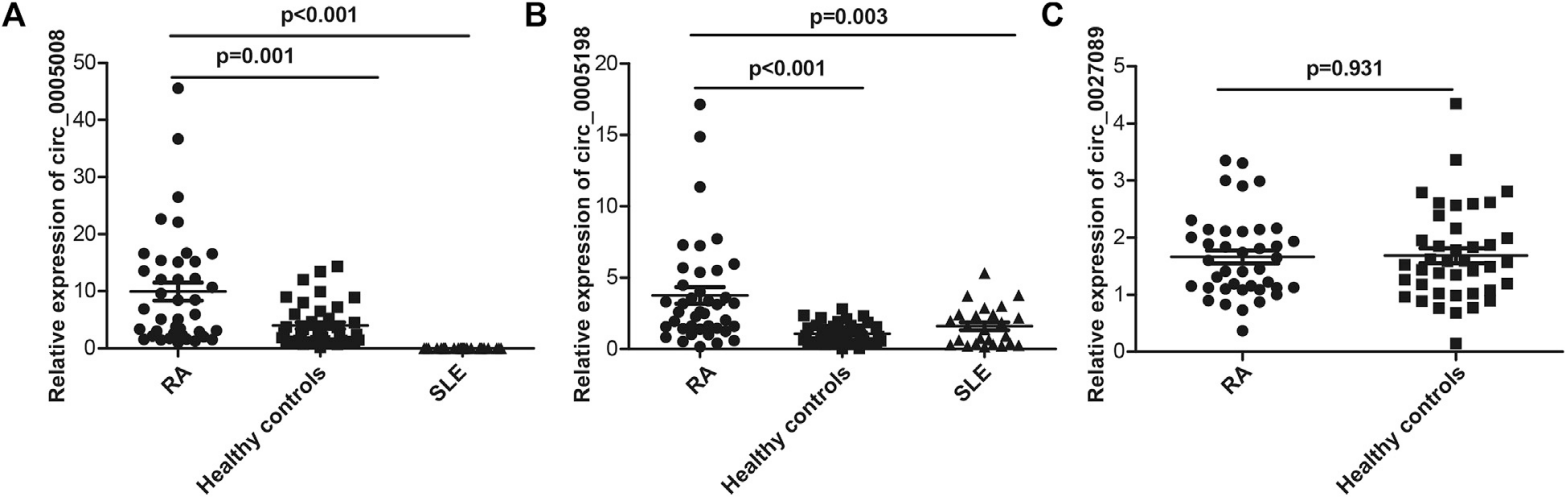
qRT-PCR determination of the relative circRNA expression levels in plasma from 40 new-onset patients with RA, 40 healthy controls, and 25 new-onset patients with SLE. **(A)**: The circ_0005008 levels in plasma from new-onset patients with RA were significantly higher than those in healthy controls, and it was not expressed in the plasma of patients with SLE; **(B)**: The circ_0005198 levels in plasma from new-onset patients with RA were significantly higher than those in healthy controls and patients with SLE; **(C)**: The circ_0027089 levels in plasma were not significantly different among new-onset patients with RA and healthy controls.

Seven circRNAs that were upregulated and dysregulated according to their *p* values (<0.05), fold changes (>5), and raw intensities (>100) (circ_0001568, circ_0023990, circ_0005008, circ_0000407, circ_0005198, circ_0034642, and circ_0027089) were selected to validate the microarray expression data. An independent set of plasma samples from new-onset patients with RA and HCs (*n* = 40 for both) were subjected to qRT-PCR of the seven candidate circRNAs. The results showed significantly upregulated expression in plasma of circ_0005008 and circ_0005198 in new-onset patients with RA compared with that in HCs (Figures 2A,B), but no significant difference for circ_0027089 (Figure 2C). Circ_0001568 and circ_0034642 expression was only observed in a few participants. Circ_0000407 and circ_0015278 expression was not observed in new-onset patients with RA or HC plasma. Interestingly, the plasma expression of circ_0005198 was increased significantly in new-onset patients with RA compared with that in new-onset patients with SLE (Figure 2B). However, no expression of circ_0005008 was observed in the plasma from new-onset patients with SLE (Figure 2A).

### Testing Plasma circ_0005008 and circ_0005198 Levels and Clinical Variables in New-Onset Patients With RA Using Spearman’s Correlation

**FIGURE 3:**
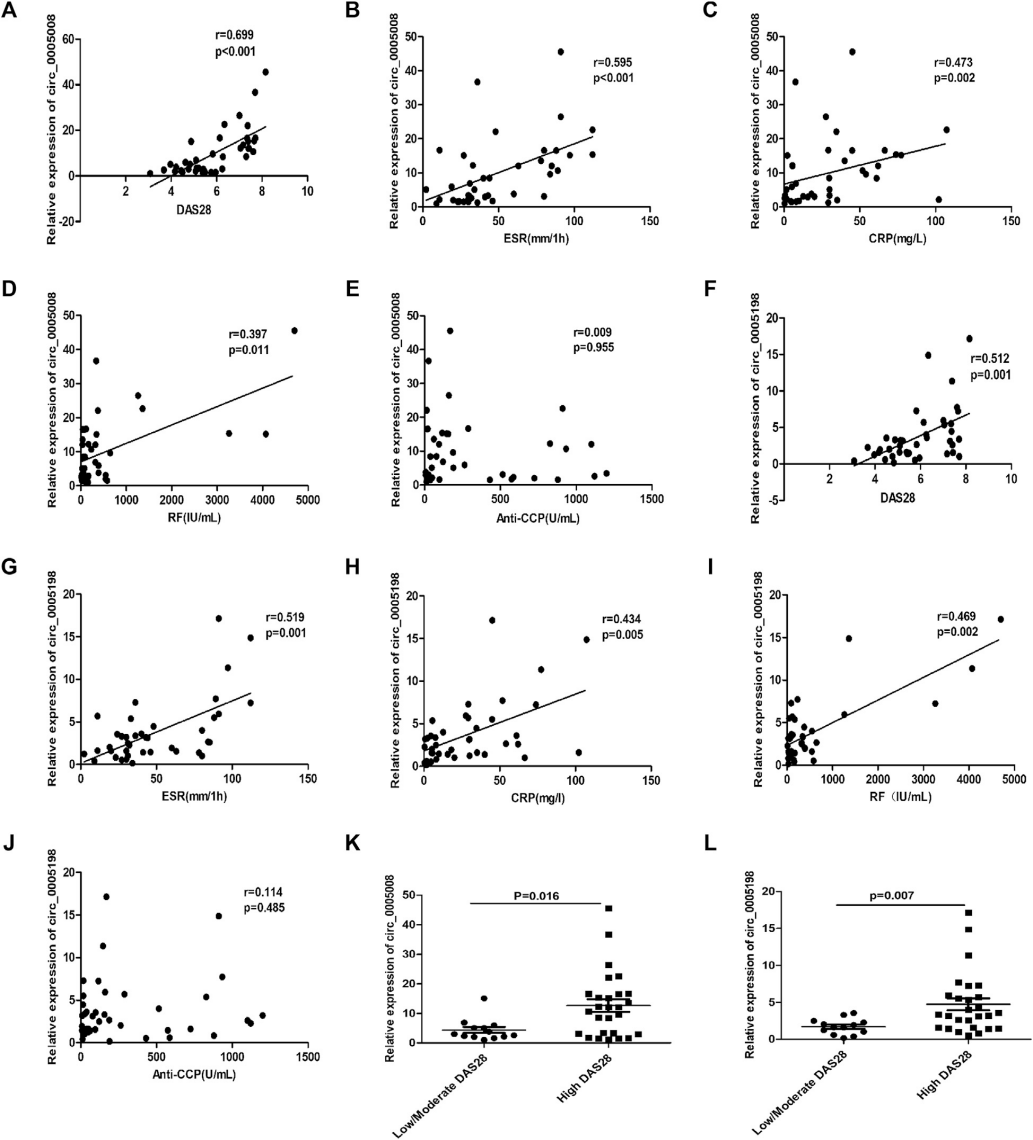
Correlations between plasma circRNA_005008 and clinical variables in new-onset patients with RA (Spearman’s rank correlation coefficients). **(A–D)** The circ_0005008 levels in plasma from new-onset patients with RA correlated positively with DAS28, ESR, CRP, and RF. **(E)**: The circ_0005008 levels in plasma from new-onset patients with RA showed no correlation with anti-CCP. **(F–I)**: The plasma level of circ_0005198 in new-onset patients with RA correlated positively with DAS28, ESR, CRP, and RF. **(J)**: The circ_0005198 levels in plasma from new-onset patients with RA showed no correlation with anti-CCP. **(K,L)**: The circ_0005008 and circ_0005198 levels in low/moderate DAS28 group (score 2.6 to ≤5.1) were significantly lower than that in high DAS28 group (score >5.1).

To determine the possibility of using the plasma expression levels of circ_0005008 and circ_0005198 in new-onset patients with RA as biomarkers for RA disease activity, Spearman’s correlation test was used to investigate the correlations between RA-associated clinical variables and circ_0005008 and circ_0005198 plasma expression levels from new-onset patients with RA. The plasma levels of circ_0005008 and circ_0005198 in new-onset patients with RA both showed positive correlations with the disease activity score in 28 joints (DAS28) (*r* = 0.699, *p* < 0.001; r = 0.512, *p* = 0.001), the erythrocyte sedimentation rate (ESR) (*r* = 0.595, *p* < 0.001; r = 0.519, *p* = 0.001), C-reactive protein (CRP) (*r* = 0.473, *p* = 0.002; *r* = 0.434, *p* = 0.005), and rheumatoid factor (RF) (*r* = 0.397, *p* = 0.011; *r* = 0.469, *p* = 0.002), respectively, all of which are indicators of disease severity. However, the expression levels of these circRNAs showed no correlation with anti-cyclic citrullinated peptide antibodies (anti-CCP) (*r* = 0.009, *p* = 0.955; *r* = 0.114, *p* = 0.458) (Figures 3A–J). In addition, we found that compared to low/moderate DAS28 group, the expression of circ_0005008 and circ_0005198 were significantly increased in high DAS28 group (Figures 3K,L).

### ROC Curve Analysis of Circ_0005008 and Circ_0005198 Levels in Plasma From New-Onset Patients With RA

**FIGURE 4:**
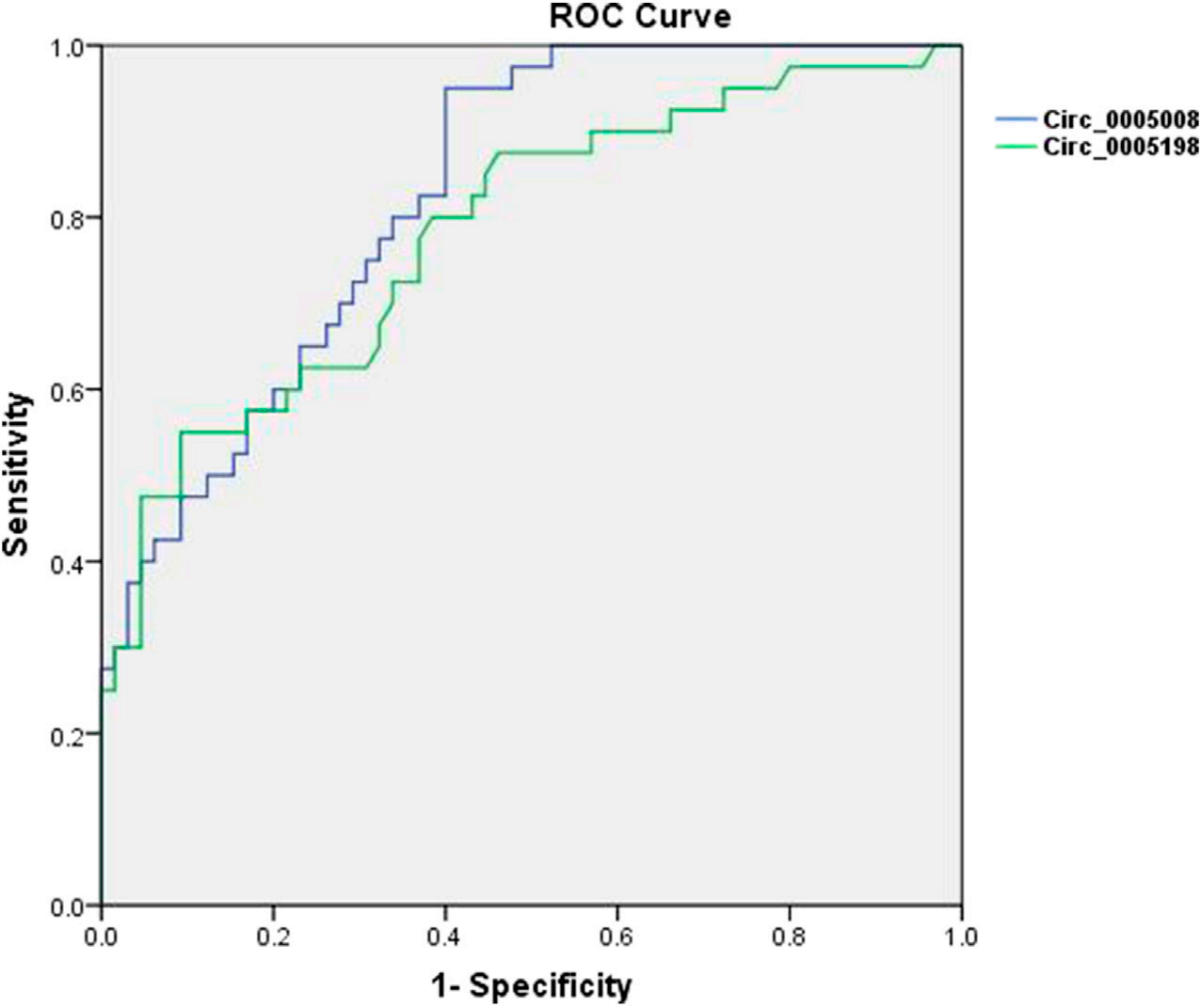
ROC curves for plasma circ_0005008 and circ_0005198 in patients with RA. The AUC of plasma circ_0005008 was 0.829 (95% CI 0.754–0.904, *p* < 0.001) and 0.783 (95% CI 0.690–0.875, *p* < 0.001).

To additionally test the potential validity of circ_0005008 and circ_0005198 as diagnostic biomarkers of RA, ROC curve analysis was applied. When distinguishing new-onset patients with RA from controls (patients with SLE and HCs), the areas under the ROC curve (AUCs) for circ_0005008 and circ_0005198 were 0.829 and 0.783, respectively. The optimal cut-off value for circ_0005008 and circ_0005198 were 1.457 and 2.454, respectively. The diagnostic sensitivities were 0.950 for circ_0005008 and 0.550 for circ_0005198, and their specificities were 0.600 and 0.908, respectively. The positive predictive values (PPV) for circ_0005008 and circ_0005198 were 0.594 and 0.876, and their negative predictive values (NPV) were 0.951 and 0.766 (Figure 4).

### Circ_0005198 and miR-4778-3p Expression Levels Correlated Negatively in RA-FLSs

**FIGURE 5:**
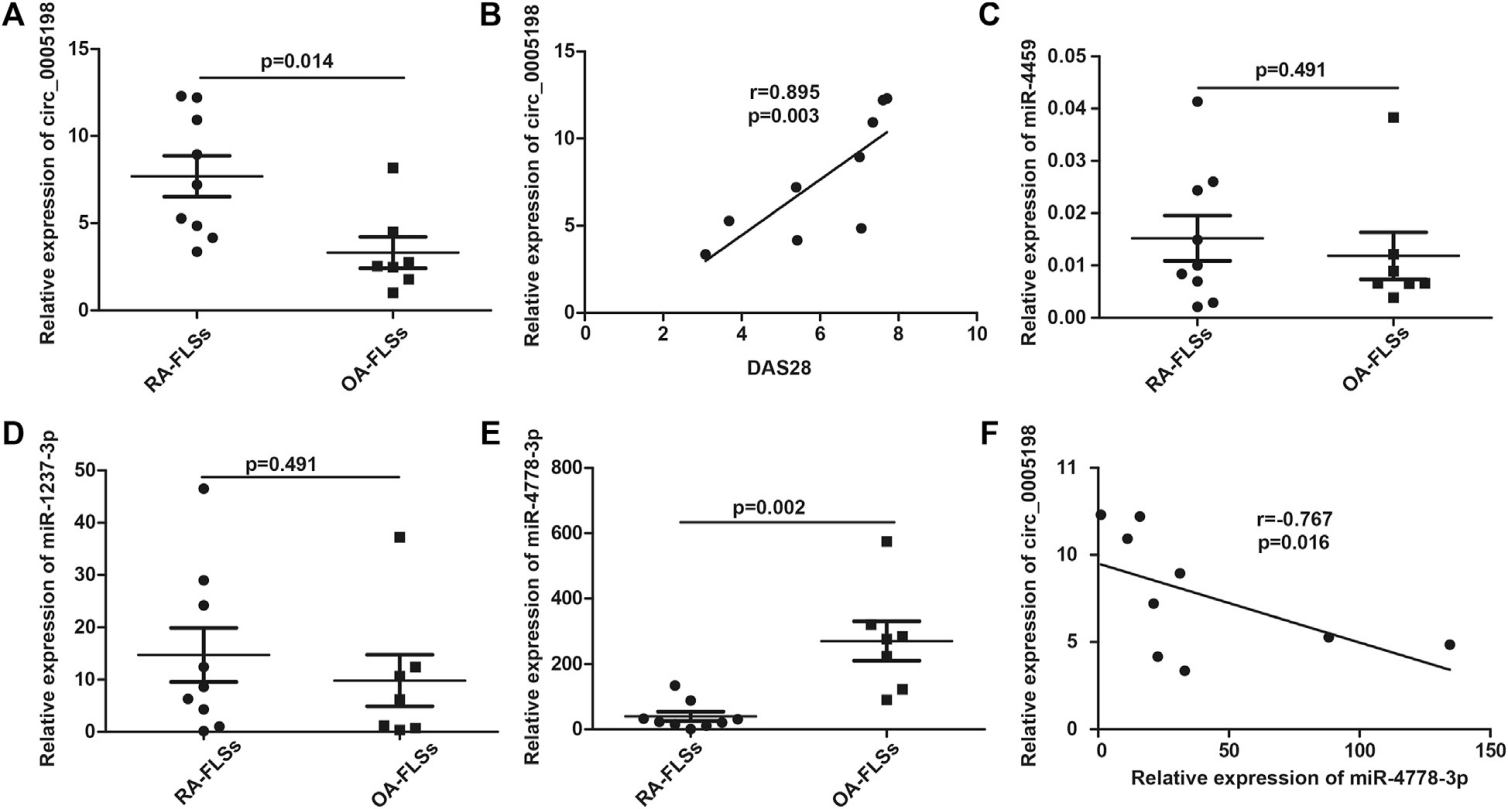
qRT-PCR determination of the relative circ_0005198 expression and its potential target miRNAs in RA-FLSs and OA-FLSs. **(A)**: The level of circ_0005198 in OA-FLSs was significantly lower than that in RA-FLSs; **(B)**: The level of circ_0005198 in RA-FLSs correlated with DAS28; **(C,D)**: The level of miR-4459 and miR-1237-3p were not significantly different among RA-FLSs and OA-FLSs; **(E)**: The RA-FLSs level of miR-4778-3p was decreased significantly compared with its OA-FLSs level; **(F)**: The level of circ_0005198 in RA-FLSs correlated negatively with the level of miR-4778-3p.

Circ_0005198 expression was upregulated significantly in RA-FLSs compared with that in OA-FLSs (Figure 5A), and its expression level correlated positively with the DAS28 (Figure 5B). However, the expression level of circ_0005008 was not detectable in RA-FLSs. To determine the potential functions of circ_0005198, Arraystar’s miRNA target prediction software was used to align the microRNA response elements (MREs) of differentially expressed circRNAs with miRNAs to predict their target miRNAs. Five potential miRNA targets (miR-4459; miR-450b-5p; miR-4254; miR-4778-3p; and miR-1237-3p) were identified for circ_0005198. However, we observed no significant differences in the levels of miR-4459 and miR-1237-3p between RA-FLSs and OA-FLSs (Figures 5C,D); however, in RA-FLSs, the level of miR-4778-3p decreased significantly compared with that in OA-FLSs (Figure 5E). In addition, the expression levels of circ_0005198 and miR-4778-3p correlated significantly and negatively (Figure 5F), suggesting that circ_0005198 might exert its function in RA-FLSs by interacting with miR-4778-3p. However, the levels of miR-450b-5p and miR-4254 in RA-FLSs were not detectable.

## Discussion

Recent studies have shown that abnormal expression of circRNAs could be used as novel biomarkers for several autoimmune disease, such as RA (Luo et al., 2018) and SLE (Guo et al., 2019). Our previous study showed that the plasma level of circRNA_002453 could be used as a novel biomarker to diagnose lupus nephritis (Ouyang et al., 2018) and the circRNAs present in patients with RA’s peripheral blood mononuclear cells might be used as novel biomarkers for RA (Ouyang et al., 2017). However, the role of circRNAs in plasma from new-onset patients with RA is unclear. In the present study, microarray analysis was performed to compare the expression profiles of plasma circRNAs from new-onset patients with RA and those from HCs, which identified dysregulated (significantly differentially expressed) circRNAs. Thus, the results of our study might provide useful data for the pathophysiological research into RA and could answer the question as to whether plasma levels of circRNAs have utility as non-invasive biomarkers to diagnose RA.

In the present study, circRNA microarray profiling followed by qRT-PCR validation identified significantly higher expression levels of circ_0005008 and circ_0005198 in new-onset patients with RA than in HCs and new-onset patients with SLE. This indicated that plasma circ_0005008 and circ_0005198 might function in RA pathogenesis. In addition, the plasma expression levels of circ_0005008 and circ_0005198 in new-onset patients with RA both correlated with DAS28, ESR, CRP, and RF, which reflect the severity of disease activity (Atzeni et al., 2017; Zhang et al., 2018). Thus, plasma levels of circ_0005008 and circ_0005198 might serve as biomarkers for RA systemic inflammation and disease severity. Notably, their expression levels did not correlate with anti-CCP, which reflects the prognosis of patients with RA (Atzeni et al., 2017), indicating that plasma circ_0005008 and circ_0005198 are not potential biomarkers for patient prognosis. Moreover, the plasma levels of circ_0005008 and circ_0005198 had AUCs of 0.829 and 0.783 to discriminate new-onset patients with RA from controls (HCs and patients with SLE) and showed high specificity and sensitivity, which indicated that they have good potential as RA diagnostic biomarkers.

To further explore the role of circ_0005008 and circ_0005198 in the pathogenesis of RA, we demonstrated that the expression of circ_0005198 was significantly upregulated in RA-FLSs compared with that in OA-FLSs, and its expression level correlated positively with the DAS28. However, the expression level of circ_0005008 was not detectable in RA-FLSs. This result indicated that plasma circ_0005198 might function in RA-FLSs. Evidence has shown that circRNAs exert their biological roles by targeting and binding to miRNAs, acting as miRNA sponges (Jeck and Sharpless, 2014). Therefore, bioinformatic analysis was employed to predict the potential target miRNAs. For circ_0005198, five putative miRNAs targets (miR-4459, miR-450b-5p, miR-4254, miR-4778-3p, and miR-1237-3p) were identified. We found that the miR-4778-3p expression level was significantly lower in RA-FLSs than that in OA-FLSs, and correlated significantly and negatively with the expression level of circ_0005198. This suggested that circ_0005198 might exert its function in RA-FLSs via an interaction with miR-4778-3p. A previous study showed that miR-4778-3p could reduce the migration, proliferation, and vitality of radioresistant cervical cancer cells (Zhang et al., 2019). Therefore, we hypothesized that circ_0005198 might affect the development of RA by inhibiting the protective effect of miR-4778-3p in RA-FLSs.

However, the present study had certain limitations. First, the sample size was relatively small; therefore, these results should be validated in a larger sample and in different populations (ethnicities and regions). Second, to determine whether circ_0005008 and circ_0005198 could be diagnostic biomarkers, they should be assessed for their ability to distinguish effectively RA from other rheumatic diseases characterized by joint damage, for example ankylosing spondylitis and OA. Third, the function of circ_0005198 in RA pathogenesis was not assessed; therefore, further experiments should be performed to test the causal association between aberrant circ_0005198 expression and RA.

## Conclusion

**FIGURE 6:**
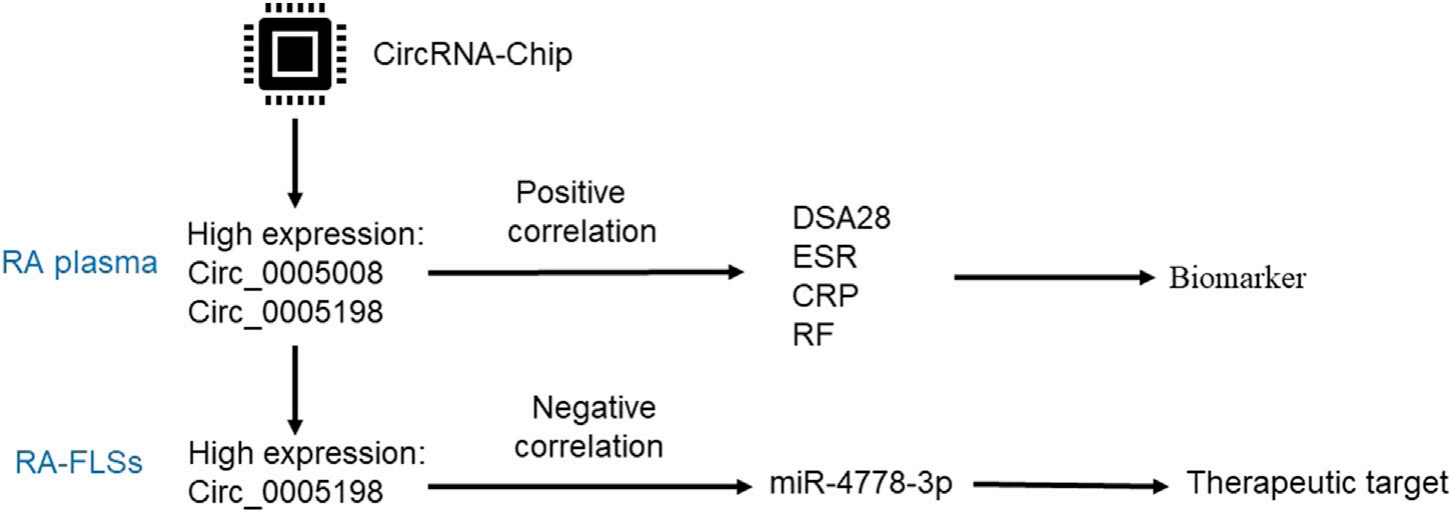
A workflow of how plasma circ_0005008 and circ_0005198 in new-onset patients with RA might serve as potential biomarkers for disease severity and diagnosis, and how circ_0005198 might function in RA-FLSs via its interaction with miR-4778-3p.

The present study identified dysregulated plasma circRNAs between new-onset patients with RA and HCs. These results will improve our understanding of the functions of circRNAs in RA. Furthermore, plasma circ_0005008 and circ_0005198 were identified as potential biomarkers of disease activity in new-onset patients with RA and might also have value in RA diagnosis. Mechanistically, in the development of RA, circ_0005198 might inhibit the protective effect of miR-4778-3p in RA-FLSs (Figure 6). These results have possible clinical applicability, and further detailed study of the biological functions and molecular mechanisms of circRNAs is warranted.

## Data Availability

The original contributions presented in the study are included in the article/Supplementary Material, further inquiries can be directed to the corresponding authors.
