# Supplementary material for: Identification of Circular RNAs Circ_0005008 and Circ_0005198 in Plasma as Novel Biomarkers for New-Onset Rheumatoid Arthritis
Source: Front Pharmacol. 2021 Sep 1;12:722017. doi: 10.3389/fphar.2021.722017 (PMC8440797; doi:10.3389/fphar.2021.722017)
Supplement: Supplementary file 1 [file DataSheet1.docx]

**Supplementary figure 1.** Fluorescence micrographs with staining for vimentin in RA-FLSs at ×200.

**
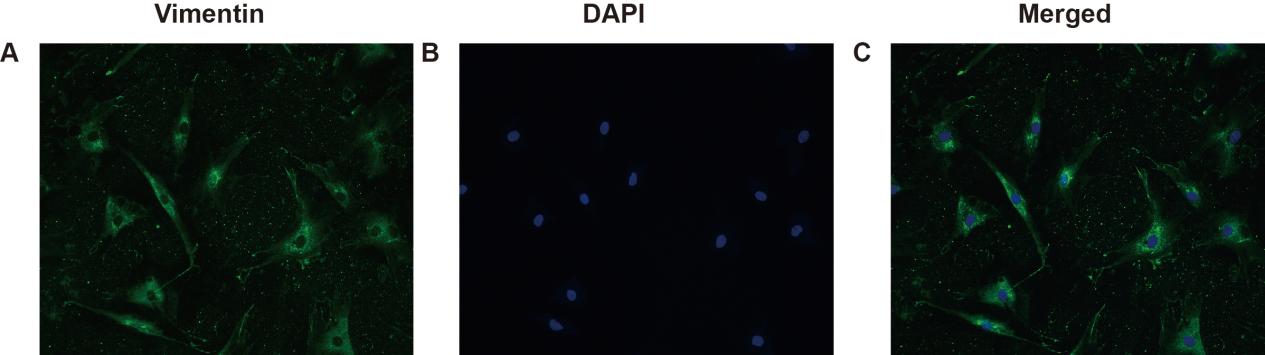
**

**Supplementary figure 2 .** Raw graphs of circRNA microarray. Five plasma samples on the above two row are HCs, five plasma samples on the bottom two row are new-onset patients with RA.


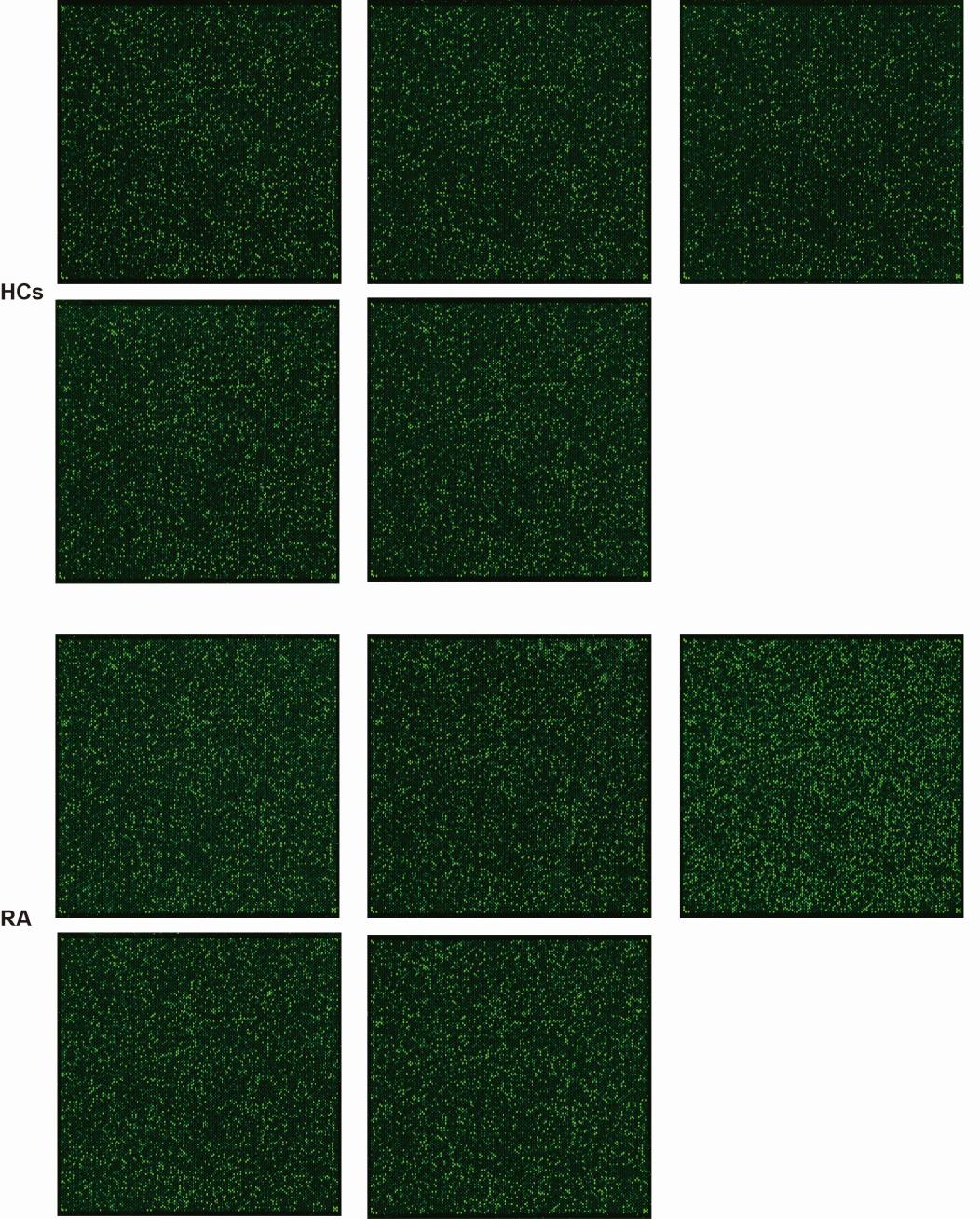


**Table S1.** Specific primers for quantitative PCR analysis.

| **Name** | **Sequence** |
| --- | --- |
| β-actin（Human） | F:5' GTGGCCGAGGACTTTGATTG3'  R :5’CCTGTAACAACGCATCTCATATT3’ |
| hsa_circ_0001568 | F:5’ GTGTGTGGGTGACTAAGTGGATG3'  R:5’ CTCAGGTGCAGCCAGGGTA 3' |
| hsa_circ_0023990 | F:5’ GGTGGTGGTGCTATTCCTCA3'  R:5’ CAATCTCCTGGTTCTCCTGCT3' |
| hsa_circ_0005008 | F:5’ CTTTGGTGGCAGCCGTGGTG 3’  R:5’GTGGTGCCGACGGTGGTTTC3’ |
| hsa_circ_0000407 | F:5’ AACGGGAGATGGTGGTGATGC 3'  R:5’ ATGGCGAACCCTCTACATCCTAAC3' |
| hsa_circ_0005198 | F:5’ CCTTGGAGAAAGTGGGAATGGA3'  R:5’ GCTTTTTCTTCTGCTGCTGAGGT 3' |
| hsa_circ_0034642 | F:5’ AGGATGGTCGGTGGTGGTGAT3'  R:5’ CTTCTGTCCATTTCGGTTCACG 3' |
| hsa_circ_0027089 | F:5’ ATGGGTGGTGA TGAGGATGTA 3'  R:5’ AGTCGACACTAAGCCAATTAAGAT3' |
| hsa-miR-4459-F | F:5’CCAGGAGGCGGAGGAGGTGGAG3'  R:Universal Reverse Primer |
| hsa-miR-450b-5p-F | F:5’TTTTGCAATATGTTCCTGAATA3'  R:Universal Reverse Primer |
| hsa-miR-4254-F | F:5’GCCTGGAGCTACTCCACCATCTC3'  R:Universal Reverse Primer |
| hsa-miR-4778-3p-F | F:5’TCTTCTTCCTTTGCAGAGTTGA3'  R:Universal Reverse Primer |
| hsa-miR-1237-3p-F | F:5’TCCTTCTGCTCCGTCCCCCAG3'  R:Universal Reverse Primer |
| U6 | F:5'GGAACGATACAGAGAAGATTAGC3'  R:5'TGGAACGCTTCACGAATTTGCG3' |
